# Combination of S100A12/TLR2 signaling molecules and clinical indicators in a new predictive model for IVIG-resistant Kawasaki disease

**DOI:** 10.1038/s41598-024-57897-z

**Published:** 2024-03-27

**Authors:** Yali Wu, Pan Liu, Yang Zhou, Youjun Yang, Shiyu Li, Wei Yin, Fan Liu, Yan Ding

**Affiliations:** 1 Department of Rheumatology and Immunology, Wuhan Children’s Hospital (Wuhan Maternal and Child Healthcare Hospital), Tongji Medical College, Huazhong University of Science and Technology, Wuhan, 430016 China; 2 Department of Pediatrics, Wuchang Hospital Affiliated to Wuhan University of Science and Technology, Wuhan, 430063 China

**Keywords:** Kawasaki disease, Intravenous immunoglobulin-resistant, S100A12, Predictive scoring model, Cardiovascular biology, Risk factors

## Abstract

Although intravenous immunoglobulin (IVIG)-resistant Kawasaki disease (KD) presents with persistent inflammatory stimulation of the blood vessels and an increased risk of coronary artery dilatation. However, the pathogenesis of this disease is unclear, with no established biomarkers to predict its occurrence. This study intends to explore the utility of S100A12/TLR2-related signaling molecules and clinical indicators in the predictive modeling of IVIG-resistant KD. The subjects were classified according to IVIG treatment response: 206 patients in an IVIG-sensitive KD group and 49 in an IVIG-resistant KD group. Real-time PCR was used to measure the expression of S100A12, TLR2, MYD88, and NF-κB in peripheral blood mononuclear cells of patients, while collecting demographic characteristics, clinical manifestations, and laboratory test results of KD children. Multi-factor binary logistic regression analysis identified procalcitonin (PCT) level (≥ 0.845 ng/mL), Na level (≤ 136.55 mmol/L), and the relative expression level of S100A12 (≥ 10.224) as independent risk factors for IVIG-resistant KD and developed a new scoring model with good predictive ability to predict the occurrence of IVIG-resistant KD.

## Introduction

Kawasaki disease (KD) is an immuno-inflammatory disease occurring mostly in children < 5 years and presents with characteristic systemic small- and medium-sized vasculitis^1^. Approximately 25% of untreated patients develop coronary artery lesions (CALs)^2^, including coronary artery dilation and coronary aneurysms, potentially leading to thrombotic stenosis, embolism, and even sudden death^3^. The typical treatment for this disease is a single intravenous injection of immunoglobulin (IVIG) at a dose of 2 g/kg in combination with oral aspirin, which reduces the chances of coronary artery damage to 5%^2^. Moreover, the incidence of coronary artery damage is high in patients with IVIG resistance^4^, with approximately 10–20% of patients experiencing coronary artery damage^5^. Thus, this condition requires clinician attention and effective treatment to resolve the inflammatory process and improve patient prognosis. However, the pathogenesis of IVIG-resistant KD is unclear, and no specific biomarkers are currently established to predict the risk of this disease. Therefore, in this study, we combined S100A12/TLR2-related signal transduction molecules and clinical indicators to construct a prediction model for IVIG-resistant KD as well as to decipher its pathogenesis and help in the clinical decision-making in this disease.

## Materials and methods

### Study population

Study cohort: Children with KD admitted to our rheumatology department from January 2022 to December 2022 and having complete medical history and test and examination results were included in the cohort population. The patients were categorized into an IVIG-sensitive or -resistant KD group according to their response to the initial IVIG administration.

Inclusion criteria: (i) patients meeting the 2017 diagnostic criteria for KD published by the American Heart Association^2^, (ii) complete data, (iii) age < 18 years, (iv) first diagnosis of KD, and (v) hospital admittance within 10 days of fever and treatment with single IVIG (2 g/kg) and oral aspirin (30–50 mg/kg/day).

Exclusion criteria: (i) patients with incomplete data, (ii) diagnosis of basal coronary artery disease, (iii) hormonal drug use within the last 4 weeks, (iv) KD with complications such as shock syndrome and macrophage activation syndrome, (v) secondary infections, (vi) lost to follow-up, or (vii) immunodeficiencies.

The diagnostic criteria for IVIG-resistant KD^6^ were defined as fever (temperature ≥ 38 °C) that persists 36 h after IVIG treatment or reappearance of fever, along with at least one KD symptom for 2–7 days, excluding secondary infection.

This study was approved by the institutional review board of Wuhan Children’s Hospital, Tongji Medical College, Huazhong University of Science and Technology (No. 2022R053-E01) and was conducted in accordance with Good Clinical Practice guidelines and the World Medical Association Declaration of Helsinki. The guardians of all study children provided a signed informed consent form.

**Figure 1:**
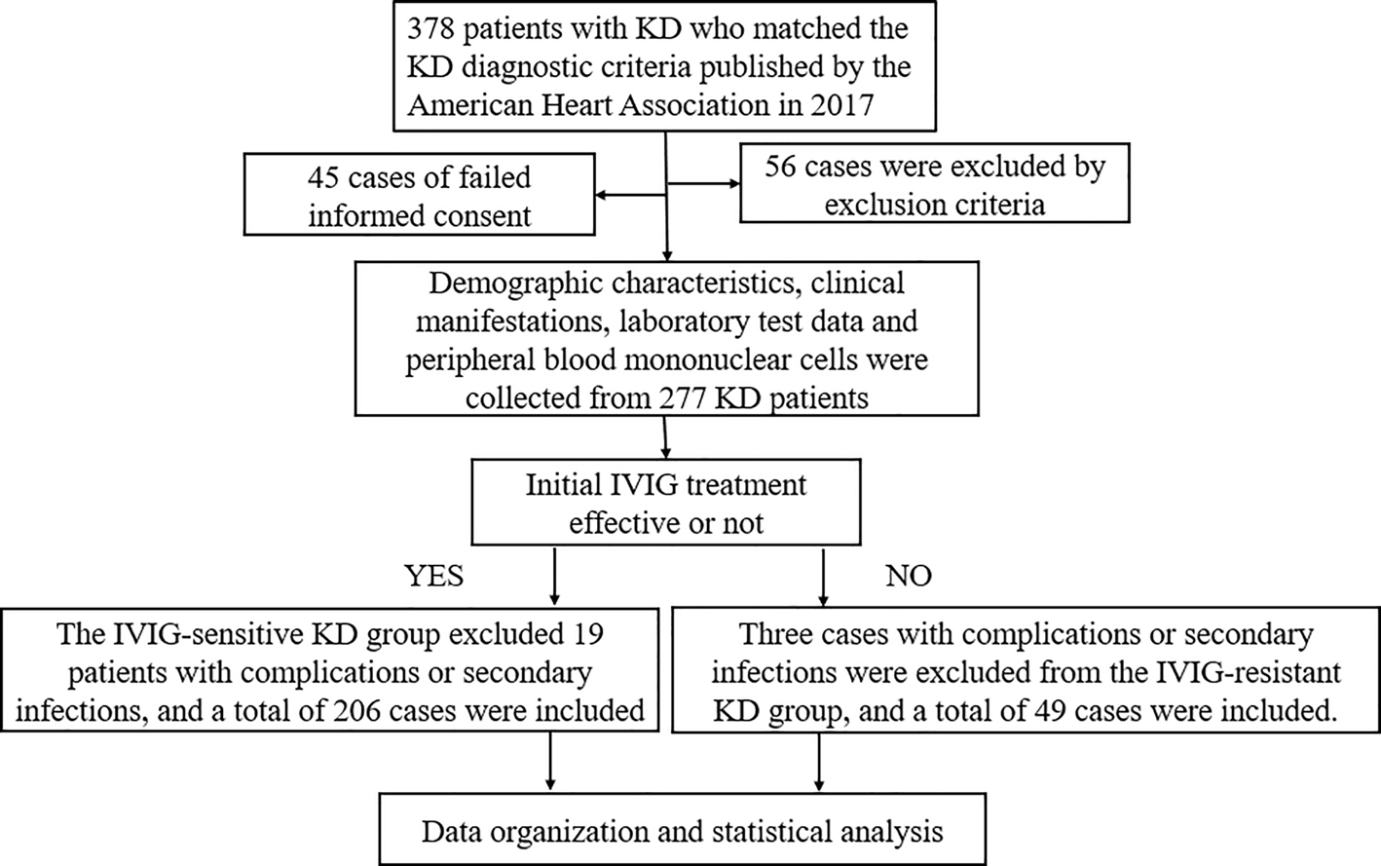
Flow chart of the patient selection process. *KD* Kawasaki disease, *IVIG* intravenous immunoglobulin.

A total of 378 cases of KD met the diagnosis criteria for KD. Among them, 56 cases were excluded based on the exclusion criteria, and 45 were excluded because their guardians did not provide signed informed consent. Consequently, 277 children with KD were finally enrolled in the study cohort. The patients were administered 2 g/kg of IVIG and oral aspirin (30–50 mg/kg/day), followed by adjusting the aspirin dose to 3–5 mg/kg/day after 48–72 h of temperature normalization. After the first IVIG treatment, patients exhibiting a response to the treatment were included in the IVIG-sensitive KD group, and those failing to respond were categorized in the IVIG-resistant KD group. Additionally, 22 cases were excluded from the study due to complications, secondary infections, or other reasons during the disease course. Ultimately, 206 cases with IVIG-sensitive KD and 49 with IVIG-resistant KD were included in the study (Fig. 1).

### Data collection

Information on all 255 patients was obtained from the electronic medical record system and data platform of Wuhan Children’s Hospital, Tongji Medical College, Huazhong University of Science and Technology. The following data were entered into an EXCEL sheet. (i) General patient information including gender, age, and hospitalization duration. (ii) Clinical manifestation data such as fever, rash, conjunctivitis, lip and tongue changes, lymphadenopathy, changes in extremities, and pyuria. (iii) Laboratory test results comprising values of white blood cell (WBC), platelets (PLTs), hemoglobin (Hb), neutrophil counts, lymphocyte counts, neutrophil-to-lymphocyte ratio (NLR = neutrophils [× 10^9^/L]/lymphocytes [× 10^9^/L]), C reactive protein (CRP), procalcitonin (PCT), erythrocyte sedimentation rate (ESR), serum ferritin (SF), alanine aminotransferase (ALT), aspartate transaminase (AST), lactate dehydrogenase (LDH), creatine kinase-MB (CK-MB), serum albumin (ALB), globulin (Glo), total bilirubin (TBIL), direct bilirubin (DBIL), serum creatinine (Scr), urea nitrogen (BUN), serum sodium (Na), serum chloride (Cl), CD3^+^CD4^+^ T cells, CD3^+^CD8^+^ T cells, CD19^+^ B cells, IgM, IgG, IgA, IgE, IL-2, IL-4, IL-6, IL-10, IL-17A, IFN-γ, and TNF-α. (iv) Cardiac ultrasound results indicating CALs or not.

Isolation of peripheral blood mononuclear cells (PBMCs): Peripheral blood was collected from all included cases with KD on admission with an anticoagulation tube, processed with Ficoll lymphocyte isolate, and the resulting solution was next placed in a horizontal centrifuge and centrifuged to extract PBMC. The separated PBMCs were washed twice with sterile phosphate-buffered saline. The cells were then resuspended in RPMI-1640 culture medium and stored at 4 °C as a reserve.

Reverse transcription-PCR (RT-PCR) was performed to detect the expression of S100A12, TLR2, myeloid differentiation primary response gene 88 (MYD88), and NF-κB in the PMBCs. The primers were designed using Primer 5.0 software and synthesized by Sangon Biotech (Table 1 displays the sequences of the used primers). Initially, RNA samples were extracted from the PMBCs according to the RT kit instructions. Next, cDNA was synthesized using reverse transcriptase. Real-time quantitative PCR was then performed using cDNA as a template, wherein the gene expression results were calculated using the 2^−ΔΔCT^ method.

**Table 1:** The sequence of primers.

Primer	Sequence
GAPDH Forward	GCACCGTCAAGGCTGAGAAC
GAPDH Reverse	TGGTGAAGACGCCAGTGGA
S100A12 Forward	AGCATCTGGAGGGAATTGTCA
S100A12 Reverse	GCAATGGCTACCAGGGATATGAA
TLR2 Forward	ATCCTCCAATCAGGCTTCTCT
TLR2 Reverse	GGACAGGTCAAGGCTTTTTACA
MYD88 Forward	GGCTGCTCTCAACATGCGA
MYD88 Reverse	CTGTGTCCGCACGTTCAAGA
NF-κB Forward	AACAGAGAGGATTTCGTTTCCG
NF-κB Reverse	TTTGACCTGAGGGTAAGACTTCT

### Statistical analysis

All data analyses were performed using SPSS 22. Normally distributed continuous variables were expressed as mean (${\overline{\text{x}}}$) ± standard deviation (s), with two independent samples *t*-test for between-group comparisons. Non-normally distributed measures were represented as median (M) (quartile 1 [Q1], quartile 3 [Q3]), and the Mann–Whitney *U*-test was used for the group comparisons. Count data were presented as the number of patients, using a four-compartment table χ^2^ test for between-group comparisons. *P* < 0.05 was considered statistically significant. The variables that showed significant differences between the two groups were subjected to one-way logistic analysis. Furthermore, a multi-factor logistic model using a *P*-value of < 0.15 was employed to screen for valuable risk factors for regression, with scoring based on the odds ratio (OR) values to create a predictive scoring model. Additionally, the total score of each enrolled patient was pooled, and the maximum Youden index corresponding to the total score cut-off value, sensitivity, and specificity was calculated using the receiver operating characteristic (ROC) curve. Statistical significance was set at *P* < 0.05.

## Results

### General patient information and clinical phenotype of the IVIG-sensitive and -resistant KD groups

Among the 255 cases of KD, 169 were male, and 86 were female. As shown in Table 2, the IVIG-resistant and -sensitive KD groups were significantly different in the proportion of patients with coronary artery dilatation (32.7% vs. 16%, *p* = 0.004) and length of hospital stay (7 [6, 9] vs. 5 [4, 6], *p* < 0.001). However, the differences in gender, age at onset, fever, rash, conjunctivitis, lymphadenopathy, and pyuria were not significantly different between the two groups (*p* > 0.05).

**Table 2:** General and clinical presentation of the IVIG sensitive and IVIG resistant groups.

Items	Total	IVIG-sensitive KD	IVIG-resistant KD	*U*/χ2	*P*
No. of patients (%)	255 (100)	206 (80.8)	49 (19.2)		
Gender (M/F)	165/90	128/78	37/12	3.1	0.078
Age (years)	3.03 [1.67, 4.96]	3.27 [1.78, 5.23]	2.06 [1.29, 4.15]	− 2.361	0.018
Length of hospitalization (days)	5 [4, 7]	5 [4, 6]	7 [6, 9]	− 7.083	0.000
Fever (n/%)	255 (100)	206 (100)	49 (100)		
Rash (n/%)	207 (81.2)	164 (79.6)	43 (87.8)	1.718	0.190
Conjunctivitis (n/%)	225 (88.2)	182 (88.3)	43 (87.8)	0.013	0.908
Lip and tongue changes (n/%)	225 (88.2)	179 (86.9)	46 (93.9)	1.86	0.173
Lymphadenopathy (n/%)	166 (65.1)	136 (66.0)	30 (61.2)	0.401	0.527
Extremity changes (n/%)	177 (69.4)	141 (68.4)	36 (73.5)	0.47	0.493
Pyuria (n/%)	45 (17.6)	36 (17.5)	9 (18.4)	0.022	0.883
CAL (n/%)	49 (19.2)	33 (16.0)	16 (32.7)	8.16	0.004

*IVIG* intravenous immunoglobulin, *KD* Kawasaki disease, *CAL* coronary artery lesion.

### Laboratory test results of the IVIG-sensitive and -resistant KD groups

A total of 36 laboratory tests were analyzed in this study. As depicted in Table 3, significant differences were found between the IVIG-sensitive and -resistant KD groups for the Hb, PCT, SF, ALT, DBIL, Scr, Na, IL-6, and IL-10 levels (*p* < 0.05). In contrast, the values of WBC, PLT, neutrophil counts, lymphocyte counts, NLR, CRP, ESR, AST, LDH, CK-MB, ALB, Glo, TBIL, BUN, Scr, CD3^+^CD4^+^ T cell counts, CD3^+^CD8^+^ T cell counts, CD19^+^ B cell counts, IgM, IgG, IgA, IgE, IL-2, IL-4, IL-17A, IFN-γ, and TNF-α were not significantly different between the two groups (*p* > 0.05).

**Table 3:** Comparison of laboratory test results between IVIG-sensitive KD and IVIG-resistant KD groups.

Variable	IVIG-sensitive KD	IVIG-resistant KD	U/t	*P*
White blood cell (10^9^/L)	11.35 [8.65, 15.56]	11.54 [8.13, 18.2]	− 0.571	0.568
Hb (g/L)	108 [102, 116]	106 [94.5, 112]	− 2.292	0.022
PLT count (10^9^/L)	341 [277, 424.25]	293 [220.5, 463]	− 0.492	0.622
NEU count (10^9^/L)	7.29 [4.61, 10.82]	7.45 [4.83, 13.89]	− 1.090	0.276
LYM count (10^9^/L)	2.51 [1.62, 3.89]	2.41 [1.45, 3.53]	− 0.771	0.440
NLR	2.94 [1.52, 5.84]	2.68 [1.73, 7.55]	− 0.885	0.376
CRP (mg/L)	67.55 [40.33, 96.88]	65.2 [31.8, 117.5]	− 0.364	0.716
PCT (ng/mL)	0.47 [0.21, 1.29]	1.89 [0.82, 5.18]	− 5.741	0.000
ESR (mm/h)	44 [18.75, 69]	45 [16, 82.5]	− 0.383	0.702
SF (ng/mL)	148.97 [114.08, 211.12]	195.12 [114.2, 282.35]	− 2.140	0.032
ALT (U/L)	17.5 [10, 44.25]	24 [16, 60]	− 2.385	0.017
AST (U/L)	29 [21, 43]	31 [25, 55.5]	− 1.528	0.126
LDH (U/L)	290 [239.75, 390]	284 [246.5, 382.5]	− 0.318	0.751
CKMB (U/L)	25 [19, 39.25]	24 [16, 36]	− 1.222	0.222
ALB (g/L)	38.6 [35.18, 41]	36.9 [31.45, 41.7]	− 1.108	0.268
GLO (g/L)	23.15 [20, 25.8]	24.7 [18.1, 32.05]	− 1.289	0.197
TBIL (μmol/L)	6.25 [4.3, 9.1]	7.9 [4.8, 12.75]	− 1.781	0.075
DBIL (μmol/L)	2.75 [2, 3.8]	3.7 [2.3, 6.2]	− 2.509	0.012
Scr (μmol/L)	27 [22.78, 33.05]	23.7 [21.1, 27.85]	− 2.865	0.004
BUN (μmol/L)	3.1 [2.5, 3.9]	3.1 [2.6, 4.4]	− 0.926	0.354
Na (mmol/L)	137.36 ± 3.13	133.74 ± 3.26	7.213	0.000
Cl (mmol/L)	101.11 ± 2.99	99.99 ± 4.16	1.785	0.079
CD3 + CD4 + T cell (cells/μL)	852 [516.25, 1272.75]	843 [478, 1321.5]	− 0.379	0.704
CD3 + CD8 + T cell (cells/μL)	583 [332.75, 871.5]	474 [285, 945.5]	− 1.134	0.257
CD19 + B cell (cells/μL)	786.5 [512, 1232.25]	805 [373.5, 1070.5]	− 0.745	0.457
IgM (g/L)	1.01 [0.75, 1.29]	0.93 [0.66, 1.27]	− 0.373	0.709
IgG (g/L)	7.33 [5.6, 8.88]	6.89 [4.84, 13]	− 0.469	0.639
IgA (g/L)	0.82 [0.52, 1.28]	0.83 [0.41, 1.55]	− 0.248	0.804
IgE (g/L)	74.65 [29.2, 173]	113 [21.1, 246]	− 0.434	0.664
IL-2 (pg/mL)	3.3 [2.06, 4.51]	3.67 [2.59, 5.45]	− 1.101	0.271
IL-4 (pg/mL)	3.15 [2.04, 4.27]	2.95 [2.14, 3.94]	− 0.392	0.695
IL-6 (pg/mL)	75.55 [34.02, 150.69]	148.16 [43.91, 368.32]	− 2.449	0.014
IL-10 (pg/mL)	12.21 [6.97, 22.29]	28.14 [7.36, 54.9]	− 3.159	0.002
IL-17A (pg/mL)	4.21 [2.57, 6.14]	4.6 [2.12, 9.68]	− 1.182	0.237
IFN-γ (pg/mL)	3.66 [2.56, 5.74]	5.2 [2.78, 7.49]	− 1.638	0.101
TNF-α (pg/mL)	5.72 [3.77, 8.84]	4.94 [3.8, 7.24]	− 1.155	0.248

*WBC* white blood cell, *PLT* platelet, *Hb* haemoglobin, *NEU* neutrophil, *LYM* lymphocyte, *NLR* neutrophil-to-lymphocyte ratio = neutrophils (× 10^9^/L)/lymphocytes (× 10^9^/L), *CRP* C reactive protein, *PCT* procalcitonin, *ESR* erythrocyte sedimentation rate, *SF* serum ferritin, *ALT* alanine aminotransferase, *AST* aspartate transaminase, *LDH* lactate dehydrogenase, *CK-MB* creatine kinase-MB, *ALB* serum albumin, *GlO* globulin, *TBIL* total bilirubin, *DBIL* direct bilirubin, *Scr* serum creatinine, *BUN* urea nitrogen.

### S100A12, TLR2, MYD88, and NF-κB expression levels of the IVIG-sensitive and -resistant KD groups

**Figure 2:**
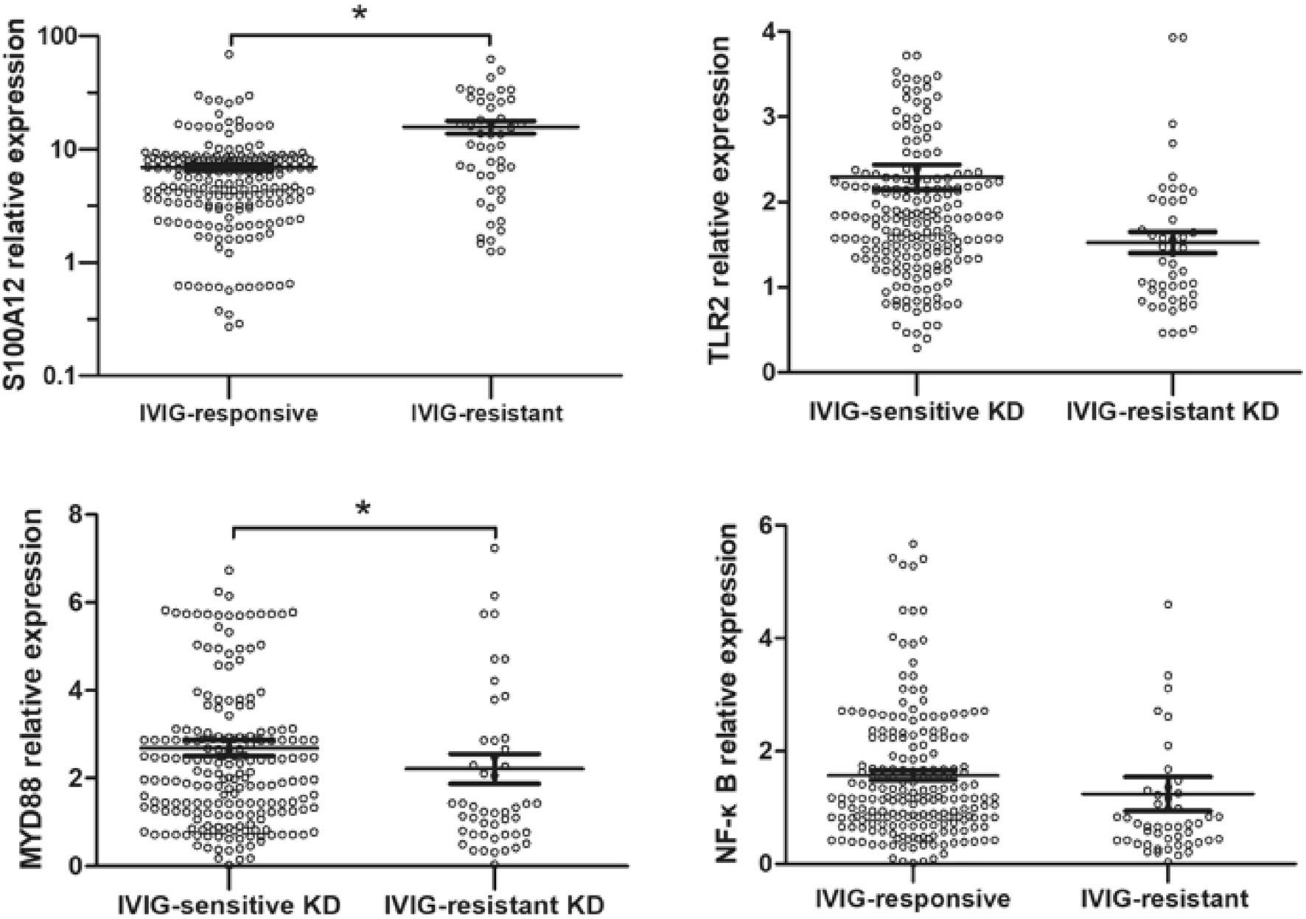
Relative expression of signaling molecules. *P < 0.05.

The relative expressions of S100A12 and MYD88 in the IVIG-resistant KD group were significantly higher than those in the IVIG-sensitive KD group (*p* < 0.05). In contrast, the differences in TLR2 and NF-κB expressions were not significantly different between the two groups (*p* > 0.05), as illustrated in Table 4 and Fig. 2.

**Table 4:** Expression of signaling molecules between IVIG-sensitive KD and IVIG-resistant KD groups.

Variable	IVIG-sensitive KD	IVIG-resistant KD	U	*P*
S100A12	5.95 [3.29, 8.39]	11.03 [4.37, 26.38]	− 4.159	0.000
TLR2	1.83 [1.39, 2.32]	1.58 [0.99, 2.17]	− 1.840	0.066
MYD88	2.13 [1.23, 3.08]	2.86 [1.39, 5.39]	− 2.353	0.019
NFKB	1.18 [0.76, 2.24]	1.22 [0.62, 2.68]	− 0.131	0.895

### Independent risk factors for the development of IVIG-resistant KD

The 11 independent variables exhibiting significant differences between the IVIG-sensitive and -resistant KD groups (*p* < 0.05) were further subjected to one-way logistic regression analysis. The analysis revealed that ALT and Scr levels were not significantly associated with the occurrence of IVIG-resistant KD (*p* > 0.15), resulting in nine independent variables, i.e., Hb, PCT, SF, DBIL, Na, IL-6, and IL-10 levels and S100A12 and MYD88 expression levels. Furthermore, multi-factor binary logistic regression analysis was conducted, ultimately identifying PCT and Na levels and S100A12 expression level as independent predictors of IVIG-resistant KD (Table 5).

**Table 5:** Logistic regression analysis to identify independent factors predicting IVIG-resistant KD.

Variable	Univariate		Multivariate	
	Crude OR (95% CI)	*P*	Adjusted OR (95% CI)	*P*
Hb (g/L)	0.968 [0.941, 0.995]	0.022		
PCT (ng/mL)	1.301 [1.151, 1.469]	0.000	1.221 [1.057, 1.412]	0.007
SF (ng/mL)	1.001 [1.000, 1.003]	0.061		
ALT (U/L)	1.000 [0.996, 1.004]	0.993		
DBIL (μmol/L)	1.016 [0.999, 1.034]	0.066		
Scr (μmol/L)	0.978 [0.941, 1.016]	0.246		
Na (mmol/L)	0.713 [0.636, 0.799]	0.000	0.729 [0.641, 0.829]	0.000
IL-6 (pg/mL)	1.000 [1.000, 1.001]	0.071		
IL-10 (pg/mL)	1.007 [1.000, 1.014]	0.066		
S100A12	1.094 [1.054, 1.135]	0.000	1.079 [1.035, 1.124]	0.000
MYD88	1.116 [1.002, 1.244]	0.047		

### Development of a scoring model for predicting IVIG-resistant KD

**Figure 3:**
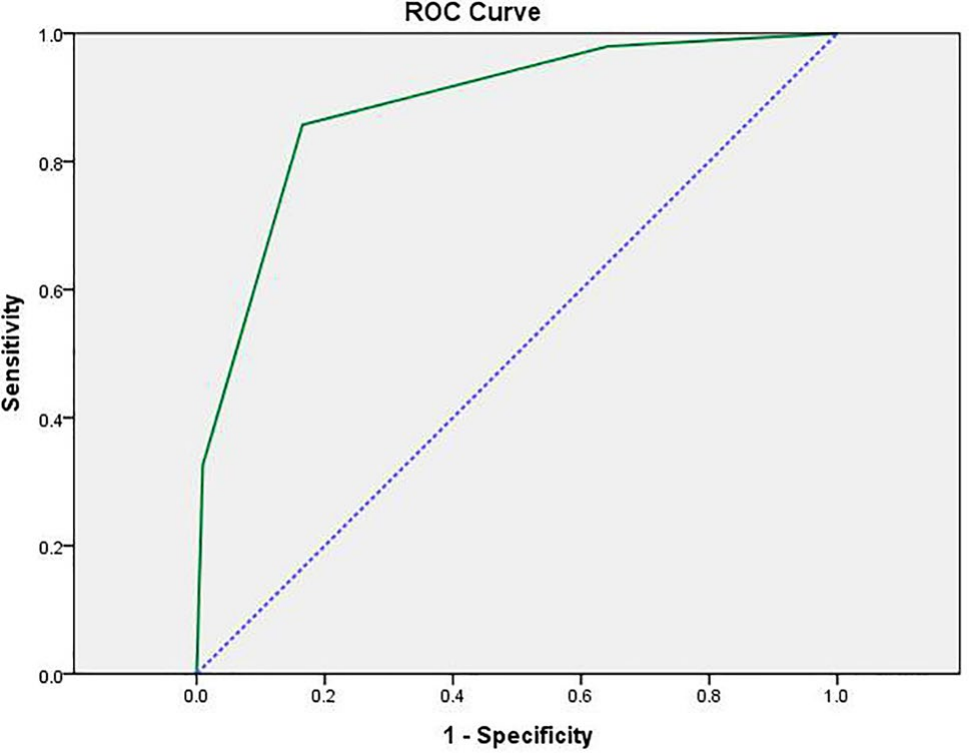
ROC curves for predicting IVIG-resistant KD using the new scoring model. AUC: 0.886 (95% CI, 0.834–0.938). *ROC* receiver operating characteristic, *IVIG* intravenous immunoglobulin, *KD* Kawasaki disease, *AUC* area under ROC curve, *CI* confidence interval.

The independent risk factors PCT and Na levels and S100A12 expression level underwent ROC analysis separately to determine their cut-off values. The values were then converted to dichotomous variables, and a scoring system was established based on the OR values (95% confidence interval [CI]). The scoring system was defined as follows: PCT level ≥ 0.845 ng/mL (1 point), Na level ≤ 136.55 mmol/L (1 point), and S100A12 expression level ≥ 10.224 (1 point). The total score was calculated for each patient, and ROC analysis cut-off values were estimated. A score of < 1.5 was classified as low risk and ≥ 1.5 as high risk for IVIG-resistant KD. In this study population, the scoring system had a sensitivity of 0.857, specificity of 0.835, Youden index of 0.692, and AUC of 0.886, along with a plotted ROC curve, as shown in Table 6 and Fig. 3.

**Table 6:** Cut-off values and points after continuous variables are converted to dichotomous variables.

Variable	Cut-off point	Sensitivity	Specificity	OR (95% CI)	*p*	Point
PCT (ng/mL)	0.845	0.755	0.66	1.221 [1.057, 1.412]	0.007	1 (≥ 0.845), 0 (< 0.845)
Na (mmol/L)	136.55	0.837	0.636	0.729 [0.641, 0.829]	0.000	1 (≤ 136.55), 0 (> 136.55)
S100A12	10.224	0.571	0.888	1.079 [1.035, 1.124]	0.000	1 (≥ 10.224), 0 (< 10.224)

### Comparison of the predictive efficacy of the new scoring system with two commonly used scoring systems

**Figure 4:**
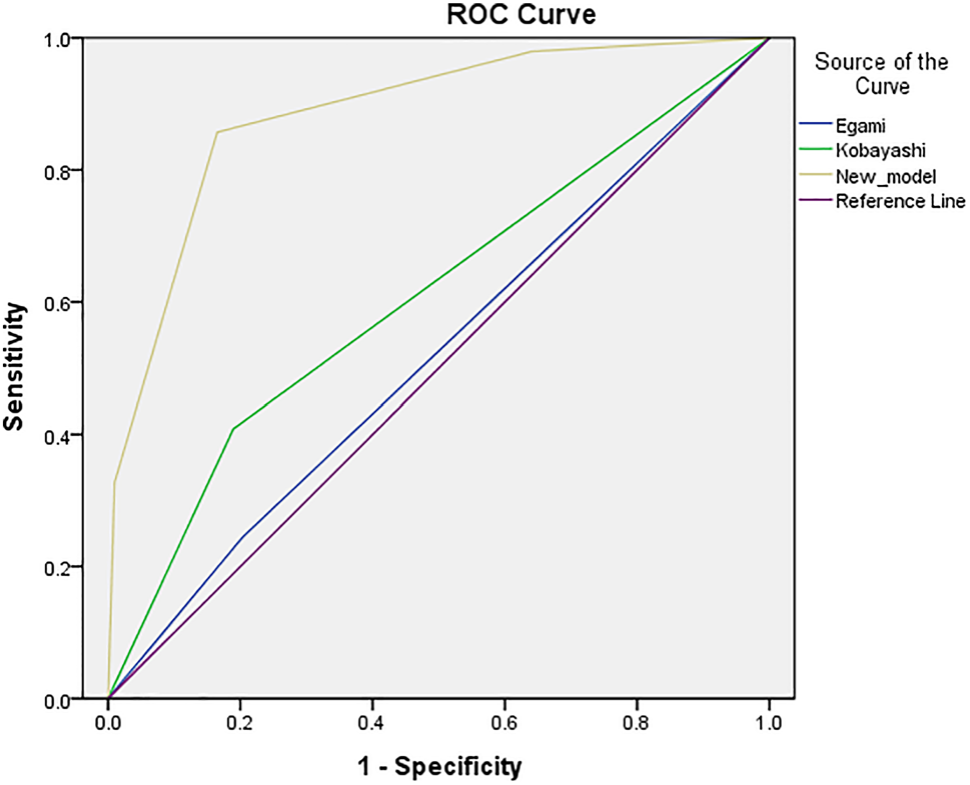
ROC curves of the three scoring models in predicting IVIG-resistant KD in subjects. AUC of the Kobayashi model: 0.609 (95% CI 0.517–0.702). AUC of the Egami model: 0.521 (95% CI 0.429–0.612). *ROC* receiver operating characteristic, *IVIG* intravenous immunoglobulin, *KD* Kawasaki disease, *AUC* area under ROC curve, *CI* confidence interval.

The Kobayashi and Egami scoring model were used to score the prediction of each variable in all the KD cases in our group and the total score was calculated for each subject, greater than or equal to the high-risk value was considered as positive (IVIG-resistant KD) and otherwise negative (IVIG-sensitive KD). The sensitivity, specificity, positive predictive value, negative predictive value and Yuden’s index of the prediction of each scoring system in this group of subjects were calculated and compared with the new scoring model (Table 7), and the ROC curves of the three scoring systems in this group of subjects were also plotted (Fig. 4). The new model had good predictive efficacy in the subjects of this study.

**Table 7:** Comparison of the predictive efficacy of three scoring models in subjects.

Scoring model	High-risk value	Sensitivity	Specificity	PPV	NPV	Youden index
Kobayashi score	≥ 4 points	0.408	0.811	0.339	0.852	0.219
Egami score	≥ 3 points	0.245	0.796	0.222	0.816	0.041
New score	≥ 1.5 points	0.857	0.835	0.553	0.961	0.692

*PPV* positive predictive value, *NPV* negative predictive value.

## Discussion

Although the pathogenesis of IVIG-resistant KD remains unclear, damage-related molecular patterns (DAMPs), which act via pattern recognition receptors, are considered a potential initiating factor in this disease pathogenesis^7,8^. S100A12, a member of the S100 family of calcium-binding proteins, is one such DAMP molecule that binds to various receptors in the extracellular environment and activates inflammatory responses^9^. The S100 family of heterodimers consisting of S100A8 and S100A9 proteins, also known as calprotectins, has been shown in many studies to serve as inflammatory biomarkers and to be of clinical value^10^. S100A12 has been reported to be secreted by neutrophils in the early stages of KD^11^ and is involved in the pathophysiological process of this disease^12^, wherein it synergistically acts to activate endothelial cells and promote CALs^13,14^. Moreover, the in vivo action of S100A12 is strictly linked with pattern recognition receptors such as TLRs^15,16^. Several studies have found that TLR2 is highly expressed in patients with KD, with Lin et al. demonstrating enhanced TLR2 expression in the monocytes of cases with KD and mouse models of coronary arteritis^17^. Similarly, a study by Kang et al. found that high TLR2 expression in monocytes was associated with CALs and IVIG resistance in KD^5^, indicating that TLR2 may be a predictor of CAL development and IVIG resistance in patients with KD^5,18^. In addition, the high expression of TLRs mainly activates the signaling mediators MYD88 and TIR domain-containing adapter-inducing interferon-β (TRIF), ultimately leading to NF-κB activation and secretion of pro-inflammatory cytokines, such as TNF-α, IL-1, and IL-6^19,20^. Furthermore, Rosenkranz et al. demonstrated that TLR2 and MYD88 promote focal coronary arteritis induced by lactobacillus extract in a KD mouse model^21^. Additionally, Mortazavi et al. confirmed that the gene transcript levels of TLR2, 3, and 9 and MYD88 and TRIF were downregulated in patients with KD after IVIG treatment^22^. Previous studies have indicated NF-κB’s involvement in the development and progression of KD via its participation in immune activation^23^ and inflammatory response as well as in the regulation of inflammatory factor release^24^ and induction of vascular endothelial damage^25^. Therefore, S100A12/TLR2 may induce increased NF-κB expression via MYD88, thereby activating the immune response against KD and potentially leading to the development of IVIG-resistant KD.

Our study found that the expressions of S100A12 and MYD88 in the IVIG-resistant KD group were significantly higher than those in the IVIG-sensitive KD group (*p* < 0.05), whereas TLR2 and NF-κB expressions showed no such significant differences between the two groups. Further, the combination of clinical indicators in the multiple linear regression analysis revealed that S100A12 expression level and Na and PCT levels were independent risk factors for IVIG-resistant KD.

As mentioned earlier, Armaroli et al. identified S100A12 as a highly expressed mediator in aseptic inflammation in KD^13^. A study by Wittkowski et al. indicated that a reverse regulation of both soluble receptor for advanced glycation end products (sRAGE) and S100A12 might be a molecular mechanism promoting systemic inflammation^26^. Additionally, an integrated in-silico approach by Srivastava et al. to explore the potential biomarker genes and pathways in KD identified S100A12 as a pivotal gene with high connectivity^27^. To our knowledge, our study is the first to establish a predictive scoring model for IVIG-resistant KD by combining S100A12/TLR2 pathway signaling molecules with clinical indicators. The study results revealed that S100A12 might be an independent risk factor in IVIG-resistant KD. This finding and the research observations mentioned above confirm that S100A12 plays an important role in the pathogenesis of KD and IVIG-resistant KD, suggesting the potential use of S100A12 as a predictive biomarker for IVIG-nonresponsive KD.

Multiple studies have reported hyponatremia as a predictor of IVIG-resistant KD, with a threshold value of 133–135.35 mmol/L of Na^28–31^. This pathogenesis may be attributed to the fact that compared to IVIG-sensitive KD, IVIG-resistant KD results in increased levels of IL-6, TNF-α, and other cytokines^32^, which in turn cause excessive secretion of antidiuretic hormone^33^ that leads to increased blood volume and decreased blood Na^34^. Correspondingly, the current study also confirmed hyponatremia predicts IVIG-resistant KD. Furthermore, the critical value of 136.55 mmol/L of serum Na in the present participants was slightly higher than that in the patients from the previous study, which may be due to the geographical differences or varied duration of KD fever.

The widespread use of PCT levels in clinical settings has revealed the association of elevated concentration of PCT with immune-related diseases, such as KD, rheumatoid arthritis, and stress trauma, as well as bacterial infections^35^. Many studies have suggested using PCT levels to predict the occurrence of IVIG-resistant KD. Research investigations by Yoshikawa et al.^36^, Dominguez et al.^37^, Nakashima et al.^38^, and Nakamura et al.^39^ demonstrated that the critical value of PCT for predicting IVIG-resistant KD ranged from 0.5 to 4.3 ng/mL, confirming the crucial role of PCT in drug resistance in KD. In support of this finding, the present study also found that PCT level might serve as a predictor of IVIG-resistant KD at a critical value of 0.845 ng/mL, consistent with the previous studies.

Although there are existing IVIG resistance-KD scoring models such as those of Kobayashi and Egami’s team, the sensitivity decreases significantly in different countries and regions^40,41^, and our study similarly confirms that these two scoring models have high specificity but low sensitivity in our central population, so it is necessary to establish a scoring model that is compatible with IVIG resistance-KD in central China. Our study is the first to establish a scoring model for predicting the occurrence of IVIG-resistant KD by combining S100A12/TLR2 signaling molecules with clinical indicators. The predictive model had a scoring system comprising PCT level ≥ 0.845 ng/mL (1 point), Na level ≤ 136.55 mmol/L (1 point), and S100A12 expression level ≥ 10.224 (1 point), with a score of < 1.5 considered as low risk and ≥ 1.5 designated as high risk. Furthermore, the model exhibited good predictive efficacy, with a sensitivity of 0.857, specificity of 0.835, Youden index of 0.692, and AUC of 0.886 in the current study patients. Therefore, it is expected that the new scoring model can be utilized in clinical work for the early diagnosis of IVIG-resistant KD, and the corresponding therapeutic measures can be proposed as early as possible to reduce the related complications (Supplementary Information).

Despite the numerous studies on IVIG resistance in KD, a reliable predictor of response to IVIG treatment in patients with KD is still lacking^42,43^. Moreover, considering that many studies have utilized gene expression analysis, their results may be difficult to achieve in everyday clinical practice because the required analysis methods are available in only a limited number of research centers. Furthermore, many issues exist concerning the performance of gene expression analyses across different centers. Therefore, the expansion of this study and related assessments should include more readily available tests that examine the same pathways on a protein level. This study has several limitations that should be considered. First, all clinical indicators were collected within 24 h of admission and were not categorized according to fever duration, possibly causing bias in the results. Second, the detection of the signaling molecules was performed individually; thus, flow cytometry or protein blotting could not be performed to determine the protein levels due to the limited quantity of blood specimens. Lastly, the sample size of the single-center exploratory study and the IVIG-resistant KD group was small. Consequently, the study results may have been affected by the population composition and controlled conditions in the laboratory. Therefore, further evaluation in prospective studies should include a multicenter approach and larger sample sizes to establish a validation set.

In conclusion, our study revealed that S100A12 is highly expressed in patients with IVIG-resistant KD and may be involved in its pathophysiological process. Furthermore, we established a new predictive scoring model for IVIG-resistant KD with good predictive efficacy.

### Supplementary Information

Supplementary Information 1.

Supplementary Information 2.

## Data Availability

The original contributions presented in the study are included in the article, further inquiries can be directed to the corresponding author.
